# Metagenome-Assembled Genome Sequence of *Sulfuricurvum* sp. Strain IAE1, Isolated from a 4-Chlorophenol-Degrading Consortium

**DOI:** 10.1128/MRA.00296-19

**Published:** 2019-08-01

**Authors:** Xiuying Li, Yi Yang, Xiangfeng Zeng, Jingjing Wang, Huijuan Jin, Zhi Sheng, Jun Yan

**Affiliations:** a Key Laboratory of Pollution Ecology and Environmental Engineering, Institute of Applied Ecology, Chinese Academy of Sciences, Shenyang, Liaoning, China; b University of Chinese Academy of Sciences, Beijing, China; University of Maryland School of Medicine

## Abstract

The draft genome sequence of *Sulfuricurvum* sp. strain IAE1 was assembled and annotated from the metagenome of an anaerobically grown 4-chlorophenol-degrading consortium. The draft genome size is 2,338,235 bp with a G+C content of 52.6%.

## ANNOUNCEMENT

Sulfur-oxidizing bacteria, such as *Sulfuricurvum*, are abundant in various environments and have essential roles in global sulfur cycling (1, 2). Some *Sulfuricurvum* strains are capable of degrading toxic organic compounds such as polycyclic aromatic hydrocarbons and polychlorinated biphenyl (3). A metagenome data set was generated to investigate the role of *Sulfuricurvum* spp. involved in anaerobic 4-chlorophenol (4-CP) degradation.

In June 2017, top-layer sediment samples at 1.2-m depth were grabbed from a location (latitude 41°39′46″, longitude 123°6′20″) at the Xi River (Shenyang, Liaoning, China) using shovels and sealable glass containers. All sampling apparatuses were autoclaved before use. The sediment temperature and pH were 20°C and 6.6, respectively, at the sampling time. The 4-CP-degrading consortia were grown in 160-ml serum bottles filled with 100 ml defined mineral medium (4) at 30°C in the dark. 4-CP and hydrogen were provided as the carbon substrate and electron donor, respectively. The consortia were transferred (1%, vol/vol) every 2 months using presterilized, one-time-use syringes and needles. Approximately 8.4 μg DNA was extracted from 100 mg biomass using the cetyltrimethylammonium bromide method (https://1ofdmq2n8tc36m6i46scovo2e-wpengine.netdna-ssl.com/wp-content/uploads/2014/02/JGI-Bacterial-DNA-isolation-CTAB-Protocol-2012.pdf). The library was prepared using a NovaSeq 6000 S4 kit, and sequencing was performed on a NovaSeq 6000 sequencer (Illumina, Inc., San Diego, CA, USA). The sequencing yielded a total of 158,000,142 reads at a 2 × 150-bp paired-end read length. Sequences were trimmed using Trimmomatic version 0.36 (5) with default settings to remove adaptors and filter out poor-quality reads. Quality-filtered reads were assembled with MEGAHIT version 1.1.1 (6). The draft genome sequence of a *Sulfuricurvum* strain named *Sulfuricurvum* sp. strain IAE1 was binned and assembled at an average 109.9× coverage using the Rapid Annotations using Subsystems Technology (RAST) Binning Service (RBS) developed by the PATRIC Bacterial Bioinformatics Resource Center (http://patricbrc.org) (7) and was evaluated for its completeness (98%) and contamination (4.9%) using CheckM version 1.0.8 (8). This draft genome consists of 2,338,235 bp on 65 contigs (52.6% G+C content). The *N*_50_ value is 232,115 bp, and the largest contig is 752,227 bp. Gene annotation was performed using the Comprehensive Genome Analysis Pipeline with default parameters provided by PATRIC (9).

BLASTn analysis indicated that strain IAE1’s 16S rRNA gene is closely related to that of Sulfuricurvum kujiense strain DSM 16994 (GenBank accession number NR_074398) and an environmental *Sulfuricurvum* 16S rRNA gene clone, D2CL (EU498374), with sequence similarities of 97.8% and 99.9%, respectively (10). Strain IAE1’s genome harbors a *soxABXYZ* gene cluster encoding the subunits of a thiosulfate-oxidizing multienzyme complex (11, 12) and genes encoding the Ni/Fe hydrogenase metallocenter assembly proteins HypABCDEF and the HyaA and HyaB subunits of quinone-reactive Ni/Fe-hydrogenases (11, 13, 14). A BLASTp search found that the gene products mentioned above share more than 71% similarity to their homologs in *Sulfuricurvum* sp. strain MLSB (JQGL00000000) and Sulfuricurvum kujiense strain DSM 16994 (CP002355). Arsenate reductase gene *arsC*, arsenite efflux gene *arsB*, and regulatory gene *arsR*, as well as genes encoding cobalt/zinc/cadmium resistance proteins CzcCD, were also annotated, and such features may enable strain IAE1 to tolerate and detoxify heavy metals.

### Data availability.

The draft genome sequence of *Sulfuricurvum* sp. strain IAE1 has been deposited under GenBank accession number SLTI00000000. The whole-genome shotgun sequencing project has been deposited at DDBJ/ENA/GenBank under BioSample accession number SAMN11066887 and BioProject accession number PRJNA525737. The raw sequences have been deposited in the Sequence Read Archive (SRA) under the accession number SRS4850623.
